# Seeking Care at Free Episodic Health Care Clinics in Appalachia

**DOI:** 10.13023/jah.0202.07

**Published:** 2020-04-15

**Authors:** Malerie Lazar, Sandra Thomas, Lisa Davenport

**Affiliations:** University of Tennessee, Knoxville; University of Tennessee, Knoxville; University of Tennessee, Knoxville

**Keywords:** Appalachia, access to care, episodic clinics, health care, social justice

## Abstract

**Background:**

People who live in rural Appalachia experience a wide variety of problems when seeking access to health care. Health care disparities continue to be one of the most complex and prevalent problems, and many barriers exist for impoverished men and women such as a lack of education, complications with health insurance, and personal distrust of healthcare providers.

**Purpose:**

A critical gap in the literature is the unheard voice of persons in rural underserved areas. The purpose of this study was to explore the perspectives of persons in rural Appalachia who seek healthcare services at free episodic health care clinics, a common alternative source of care.

**Methods:**

In Fall 2017, a qualitative approach was used to discover the perceptions of 12 men and women in rural Appalachia who were seeking medical care at a Remote Area Medical Clinic. A transdisciplinary research group provided insight and assistance with thematic analysis in Spring 2018–Spring 2019.

**Results:**

Five overall themes emerged capturing the essence of how rural Appalachians view the experience of seeking healthcare, which include difficulties with insurance/finances, inconsistency in care, isolation in rural areas, seeking solutions, and need to feel valued.

**Implications:**

A rich description of participant experiences portrays real-life complexities for Appalachian men and women who seek healthcare. Understanding the perceptions of persons who seek healthcare and the essence of their experiences is the first step in determining future sustainable solutions for social justice.

## INTRODUCTION

The time is 7:30 a.m. on a cool, crisp September morning and hundreds of men and women wait in line to receive care at a free weekend mobile clinic. Remote Area Medical (RAM) is a nonprofit organization that offers free mobile medical clinics for underserved areas. Most clinics are set up at a local high school or other community gathering place, such as a public fairground. Clinic personnel begin setting up clinic stations and supplies on Friday and clinic services are provided on Saturday and Sunday. In order to receive services at the clinic, individuals must receive a ticket, which grants them entrance on a first come, first serve basis. Most individuals arrive at the clinic site at midnight, which is the time gates open for parking. Ticket distribution begins at 3 a.m. in the parking lot of the clinic site and doors open for patient entry at 6 a.m. After entering the clinic, patients are registered, assessed through triage, and then directed to wait in line to receive dental, vision or medical care. Most individuals wait for approximately 1 hour to receive medical services, 2 to 3 hours for vision services, and 4 to 5 hours for dental services. On this day in September, the clinic site is crowded, and patients sit shoulder-to-shoulder with other patients while they wait patiently for their service even though they are extremely tired from lack of sleep. In order to fully understand the struggles that individuals who seek health care at episodic clinics experience, a qualitative descriptive study at Remote Area Medical sites is being conducted in the Appalachian region of the U.S. Two women have requested to be a part of the study; they want to have their voices heard. They were thankful that someone was willing to listen to them about their struggles with seeking healthcare.

## BACKGROUND

Healthcare reform and policy are topics of daily debate, yet, seldom are the voices of those in poverty heard. In 2018, 38.1 million people residing in the U.S. lived below the federal poverty line and specifically the Appalachian region struggled to advance economically, with a poverty level of 13.6% compared to the national average of 11.8%.^1^ In considering healthcare access in the U.S., a critical imperative is to understand the viewpoints of people who seek health care for themselves and their family. Greater advocacy for all citizens happens when the experiences of those who do not have adequate access to health care are explored, appreciated, and valued.

In terms of financial responsibility for health care, the U.S. operates predominately on distinct public (Medicare or Medicaid) and private (employer or self-insured) sources of funding. In an attempt to provide equitable access to insurance and healthcare, the Patient Protection and Affordable Care Act (ACA) was established in 2010 to institute “shared responsibility” among government, employers, and individuals to provide healthcare services.^2^ Medicare coverage is provided through the federal government, while Medicaid is given state-by-state and has different eligibility standards throughout the country.

With the passing of the ACA in 2010, the federal government encouraged, but did not require, states to expand Medicaid coverage to any individual who falls below the federal poverty line for household income. Thirty-seven states including Washington DC adopted the expansion, while 14 states did not. Six of the 13 Appalachian states, including Tennessee, did not elect to expand Medicaid, thus minimizing access to health care for their low-income individuals residing in these states. Of the other seven states including Virginia, implementation of the expansion took between 4 and 9 years to implement, and Virginia’s Medicaid expansion was not begun until 2019; the evaluation at Remote Area Medical clinics occurred prior to expansion.^3^ While the ACA made strides toward improving healthcare access, coverage is still not complete for all individuals, with a striking 27.5 million individuals, approximately 8.5% of the U.S. population, still uninsured.^4^ Due to lack of insurance, affordable health care is hard to obtain, forcing many residents of impoverished areas in Appalachia to turn to episodic free clinics, such as RAM and Health Wagon.^5^

A review of the literature was conducted to fully understand the barriers that vulnerable populations, such as people with a low income, encounter when seeking access to health care.^6^ A noteworthy study revealed that low-income individuals have more risk factors for health disparities than any other socioeconomic group.^7^ Additionally, these low-income individuals are more likely to live in a community together, creating “vulnerable populations” that become a healthcare desert.

Three primary barriers have an effect on why low-income individuals experience difficulties in accessing health care compared to those of higher socioeconomic status.^6^ The first barrier, lack of education, strongly correlates with minimal risk perception by those with a low family income and limited health literacy.^7^ Thus, individuals do not seek adequate and appropriate care. The second barrier, complications with finances and insurance, is often due to lack of guidance for eligibility and enrollment with government subsidized Medicaid and Medicare and misunderstandings of the 2010 Patient Protection and Affordable Care Act.^8^–^10^ The final barrier, personal distrust of health providers and the healthcare system, denotes the trouble that low-income individuals have relying on and trusting a healthcare provider when they have had negative experiences while seeking care.^11^ It is noted that the majority of negative experiences that individuals experience in seeking health care stem from limited time spent with the provider and lack of integration of the determinants of health in one’s life.^11^

## METHODS

The purpose of this qualitative descriptive study was to obtain first-person accounts of healthcare experiences from residents of rural Appalachia.^12^ The study and its procedures were approved by the Institutional Review Board of the University of Tennessee, Knoxville. RAM provided a letter of support to recruit and interview participants for the study at two separate Fall 2017 clinic sites, in Southwest Virginia and Northeast Tennessee. Both clinic sites, in Southwest Virginia and Northeast Tennessee, are in counties that have a high rate of poverty, with 18.3% below the federal poverty line in Tennessee, and 26.8% below the federal poverty line in Virginia.^13^ All individuals who were interviewed resided within 100 miles of the clinic in Southwest Virginia or Northeast Tennessee. Twelve English-speaking adult men and women were recruited and interviewed. Participants chose a pseudonym or nickname to protect their privacy and confidentiality throughout the course of the interview as well as the transcription, data analysis, and reports of findings.

A volunteer stood in line for a participant while the interview was conducted to maintain the place for the individual. After informed consent was obtained, audio recorded interviews took place in a quiet private area outside the clinic, typically lasting between 30 minutes and 1 hour. Recordings were saved on an SD drive that could be removed and uploaded to a password protected computer. A general question protocol was used to structure each interview; however, the progression of questions about healthcare experiences was largely dependent on how participants responded and the need for further elaboration, in accord with the qualitative nature of the study. Participants had the opportunity to end the interview or refuse a question without penalty. A brief demographic survey was conducted at the end of the interview, and a $10 Walmart gift card was distributed to the participants.

## RESULTS

Eleven out of the twelve participants interviewed had an annual household income of ≤$30,000, which significantly falls below the national average of $63,179 according to the U.S. Census.^1^ One of the twelve participants listed an annual household income of $75,000 dollars, a distinct outlier, but stated that he was not currently employed nor had insurance, categorizing him as uninsured. Table 1 details demographic characteristics of the twelve individuals interviewed, and Table 2 lists type of health insurance by percentage.

Interviews were transcribed verbatim and were reviewed to determine accuracy of the dialect and sentence structure of the participants in an effort to preserve their cultural characteristics. Thematic analysis of the transcripts was conducted in Spring 2018–Spring 2019, with assistance of attendees of a transdisciplinary phenomenology research group (TPRG) that met on five separate occasions for line-by-line readings and discovery of themes (commonalities evident across transcripts). The perspectives of scholars from multiple disciplines contributed to the rigor, credibility and dependability of the analysis. Five prominent themes of participant healthcare experiences were identified during the data analysis process as illustrated in Table 3: Thematic Structure of Findings. Quotations of participant words are used in naming of themes.

### 1. Theme One, “These people have to choose . . . because they have to eat”

#### Difficulties with Insurance and Finances

Arguably the most prominent theme that emerged from the data was the difficulties that participants experienced with insurance and finances. People with low incomes struggle to get the care they need, and they are acutely aware that other segments of the population do not have to wait in long lines to see providers (such as the lines at RAM) or choose between seeing a healthcare provider or eating. One participant stated, “I would do everything not to go [to the doctor] because I owe so many medical bills.” She went on to convey her perception that the healthcare system doesn’t care: “I don’t think they care. They [government] wanna act like they care, but they… people right now without insurance are dying” (Lulu). Disparities resulting from lack of insurance or limited financial resources intensify a sense of powerlessness for those of low socioeconomic status. A harsh reality is the possibility of death, as expressed by the following interview excerpts:

Something needs to be done. I’ve watched my friend suffer… I mean, he’s 36 years old and he’ll be lucky if he makes it to 40. And that’s sad because he could be helped…I mean, his, if he doesn’t have insulin that’s 478 dollars a vial, he’s gonna die. So who are you to say that “We can’t see him and write him that because he doesn’t have the money to pay that” okay so you’re telling him that he has to die? I mean, who are you to say that? (Reba)

Well, you’re sad because you know that you’re sick and you need to go see the doctor. Or, your family member is sick, and they need to go, but they can’t because you can’t afford to take them. Like, with my mom, she didn’t have health insurance and she couldn’t afford to go see the doctor, so she passed away in December of a massive heart attack. (Peg)

### 2. Theme Two. “In the last 3 years, I’ve had to change doctors probably 5 times”

#### Inconsistency in Care

Lack of access to stable primary care limits continuity of care and the necessary follow-up on management of chronic health conditions. Episodic clinics such as RAM are often used as a safety net, but patients receive inconsistent care. A patient may see a provider at a clinic, yet see other providers on subsequent visits. As noted in interviews, the lack of continuity in care increases anxiety and feelings of helplessness in patients. Marie indicates, “In the last 3 years, I’ve had to change doctors probably 5 times.” Frequent change in providers creates gaps in care and evokes uncertainty, stress, and fear, as one participant suggests, “I’m always worried that I finally got comfortable with this one [provider], is she going to leave now? And that’s where my stress comes in” (Misty).

### 3. Theme Three: “You’re talking like six or seven hours away”

#### Isolation in a Rural Area

Living in remote rural areas, particularly in Appalachia, compounds the issues of inaccessibility to care. Mountainous geography and poorly maintained roads add to the challenges faced by study participants. If lucky enough to have a provider, seeing a provider usually involved greater than a 30-minute drive, which poses a difficulty for those who rely on hourly work for wages. Marie explained that the issue is also complicated by the low number of providers in her area covered by her insurance. She stated, “You don’t really have any options with them. I called [the insurance company] and they said, ‘There’s nobody in your area.’ I said, ’Not even, like two hours away?’ And they said, ‘No, you’re talking like six or seven hours away.’ And I’m, I’m not gonna drive that far for a doctor.”

### 4. Theme Four. “It’s Kinda Tough for Me”

#### Seeking Solutions

Appalachian study participants who relied on an episodic clinic for their healthcare needs felt that there was a need for healthcare reform in the U.S.—a dream that many expressed. Solely being able to understand the inequities in the healthcare system was difficult. A participant expressed his puzzlement about better controlling his health by saying:

It’s kinda tough, I mean, not knowing exactly who you want for a doctor or where you want to go to get the medical attention and things. And you know, not having a whole lot of experience with the doctors and hospitals and things, it’s kinda tough for me anyways. (Mac)

Feelings of powerlessness about finding solutions were thematic throughout each interview. “Even when you do care, like about your health and stuff, like it’s really hard sometimes to find the right ways to get help if you need help . . . I guess…you know, complicated.” (Elvis) The overwhelming feelings of disappointment in the healthcare system and the need for solutions to the current disparities were evident in each interview.

Listening to those seeking health care at free episodic clinics reveals more than just statistics about those who cannot receive adequate health care, but the feelings of helplessness, frustration, stress, and powerlessness. The study participants convey the reality of living in poverty: the difficulties with insurance and finances, the isolation of living in a rural area, inconsistency in care, and the fight to seek solutions for adequate healthcare. As one participant so clearly expressed, “I’ve always had to fight,” a statement that epitomizes the struggle of impoverished people to receive equitable health care in America. The final theme depicts what they want and need from healthcare providers.

### 5. Theme Five. “It’s the little things that make a big difference”

#### Need to Feel Valued

When they finally got to see a healthcare provider, they described “little things” that made a difference. Essentially, they want to feel valued and worthy of provider time and attention. One participant described such an interaction: “They cared. They took the time to sit down . . . the doctor came to my bedside, sat down with me and actually talked to me like I was a human being.” (Brewer).

In such experiences, provider behaviors were rather simple behaviors, including listening respectfully to the patient and conveying kindness and compassion. Participants referred to these times as being “blessed” (Misty), in contrast to prior healthcare encounters in which providers conveyed judgmental or dismissive attitudes, dispensing a prescription in a rushed manner.

Many patients expressed appreciation for Remote Area Medical and the offer of free care despite limited continuity of care. An interviewee stated:

The people are nice [at RAM]. I mean, a lot of them are volunteers, and the doctors and the dentists and all that are here on their own time, but they’re nice. They listen, they want to help. I mean, it’s limited on some of what they can do, but it’s like they care (Reba).

The perceived quality of care received at these clinics spreads quickly through word of mouth, yet each patient interviewed felt that relying on care from an intermittent source was neither dependable nor preferable, and each patient wanted to seek solutions for better health care in the future. Patients perceived care at Remote Area Medical clinics as genuinely compassionate, culturally competent, and accessible. While Remote Area Medical provides a valuable short-term service to individuals at the clinics, a more effective sustainable solution to the lack of access to adequate healthcare in rural Appalachia is imperative.

## DISCUSSION

Numerous models of care have been designed to improve healthcare access for vulnerable populations across the globe, but seldom are members of the vulnerable groups themselves asked to participate in “defining their priorities, goals, and needs.”^14^ The participants in the present study vividly articulated their needs to be valued as people and to receive consistent care from accessible, affordable providers, without “always having to fight.”

Although limited to a relatively small sample size, and one geographic region, this research offers evidence to refute some assumptions about people with low incomes. The first barrier identified in the literature review,^6^ a lack of education, was *not* found to be prominent in the narratives of those who lived and sought care in rural Appalachia. This finding is contrary to a prevalent stereotype of uneducated mountain “hillbillies.” Many participants were quite well-versed in the healthcare industry and acutely aware of the disadvantages of their positionality living in a rural area and being of low socioeconomic status.

Additionally, many participants had previous experiences with health care and were aware that it was necessary to receive ongoing care to prevent permanent debilitating health conditions and death but did not have the means in order to seek and receive reliable, affordable care. People of low income who seek health care are not mere observers of their care, but rather want to be considered full partners in their care where their feelings, perceptions, insight, and input matters. This juxtaposition is a source of frustration and helplessness for many individuals with a low income. Similarly, negative experiences with healthcare providers, such as being disbelieved when they reported pain, led them to resent the system and perceive it as uncaring.

## IMPLICATIONS

Attaining quality, affordable, and accessible primary health care would give low-income people in Appalachia the freedom to manage their health and well-being more effectively. Promising approaches for better health outcomes deserving greater consideration by policymakers include patient-centered cross-sector models of care coordination^15^ and innovative telehealth service delivery mechanisms.^16^ Likewise, appreciating and expanding the scope of practice for advanced practice nurses in primary care, particularly in rural Appalachia, will enhance care accessibility and affordability.^17^ A clarion call to action is that systemic changes need to be made on local, state, and national levels in order to improve access to care and promote social justice for all.

**SUMMARY BOX**

**What is already known on this topic?** Healthcare disparities continue to be one of the most complex and prevalent problems in Appalachia, and many barriers exist for impoverished men and women such as a lack of education, complications with health insurance, and personal distrust of healthcare providers. A critical gap in the literature is that the voices of people in rural underserved areas who seek healthcare are unheard.

**What is added by this report?** The current study provides qualitative insight into the perspectives of people in rural Appalachia who seek healthcare services at free episodic health care clinics, a common alternative source of care. Participant experiences are richly described in their own words to portray the real-life complexities for Appalachian men and women as they strive to seek health care.

**What are the implications for future research?** Understanding the perceptions of people who seek health care and the essence of their experiences is the first step in determining future sustainable solutions for social justice. Promising approaches are patient-centered cross-sector models of care coordination, innovative telehealth service delivery mechanisms, and the further expansion of advanced nursing practice roles in primary care, which deserves future research examining the overall impact on health outcomes.

## Figures and Tables

**Table 1:** Demographic Characteristics of Study Participants

Age	Gender	Highest Level of Education	Race	Hispanic/Latino ?	Annual Household Income	Marital Status	Health Insurance?	Type of Insurance
31	Female	Some college	White	No	5,000–15,000	Single	Yes	Medicaid
39	Female	Sixth grade	White	No	5,000 or less	Married	No	None
29	Female	High school	White	No	5,000 or less	Divorced	Yes	Medicaid
47	Female	Some college	White	No	15,000–30,000	Divorced	No	None
59	Female	Some college	White	No	15,000–30,000	Widowed	Yes	Medicare
42	Female	High school	White	No	5,000–15,000	Single	No	None
26	Female	High school	White	No	5,000 or less	Married	No	None
37	Male	High school	White	No	75,000+	Married	No	None
39	Female	High school	Black	No	5,000 or less	Single	Yes	Medicaid
36	Female	Bachelor's degree	White	No	15,000–30,000	Divorced	Yes	Medicare
34	Female	High school	Hispanic	Yes	5,000 or less	Divorced	Yes	Medicaid
52	Male	High school	White	No	15,000–30,000	Single	Yes	Private

**Table 2:** Health insurance coverage by percentage

Type of health insurance	%
Medicaid	33
Medicare	17
Private	8
None	42

**Table 3:**
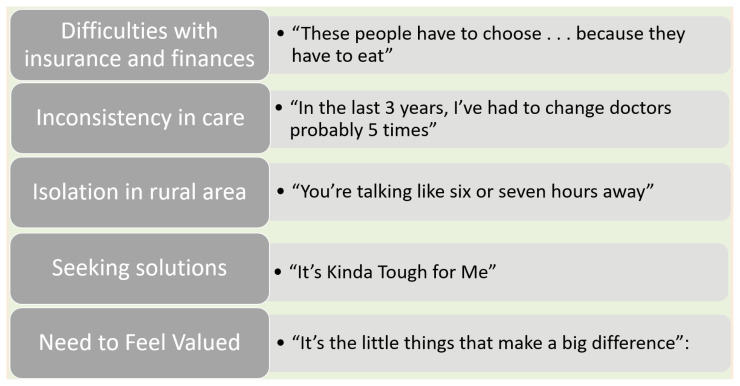
Thematic Structure of Findings

## References

[1] United States Census Bureau Income and poverty: 2018–2019 https:www.census.gov/library/publications/2019/demo/p60-266.html

[2] Commonwealth Fund The United States healthcare system 2019 https://international.commonwealthfund.org/countries/united_states/

[3] Kaiser Family Foundation 2019 Employer health benefits survey 2019 https://www.kff.org/report-section/ehbs-2019-summary-of-findings/

[4] United States Census Bureau Health insurance coverage in the United States 2018 https://www.census.gov/library/publications/2019/demo/p60-267.html

[5] Gardner T Gavaza P Meade P Adkins DM Delivering free healthcare to rural Central Appalachia population: The case of the Health Wagon Rural Remote Hlth 2012 12 1 1 7 22452285

[6] Lazar M Davenport L Barriers to health care access for low income families: a review of literature J Community Health Nurs 2018 35 1 28 37 29323941 10.1080/07370016.2018.1404832

[7] Stevens GD Seid M Mistry R Halfon N Disparities in primary care for vulnerable children: the influence of multiple risk factors Health Serv Res 2006 41 2 507 31 16584462 10.1111/j.1475-6773.2005.00498.x PMC1702517

[8] Waters EA Hay JL Orom H Kiviniemi MT Drake BF “Don’t know” responses to risk perception measures: implications for underserved populations Med Decis Making 2013 33 2 271 81 23468476 10.1177/0272989X12464435 PMC3613223

[9] Roberts ET Pollack CE Does churning in Medicaid affect health care use? Med Care 2016 54 5 483 9 26908088 10.1097/MLR.0000000000000509 PMC5548183

[b10-jah-2-2-67] 10Patient Protection and Affordable Care Act of 2010, Pub L No 111-148 Sect. 42, 2010

[11] Bolen SD Sage P Perzynski AT Stange KC No moment wasted: the primary care visit for adults with diabetes and low socio-economic status Prim Health Care Res Dev 2016 17 1 18 32 25991075 10.1017/S1463423615000134 PMC4697285

[12] Sandelowski M Whatever happened to qualitative description? Res Nsg Hlth 2000 23 334 40 10.1002/1098-240x(200008)23:4<334::aid-nur9>3.0.co;2-g 10940958

[13] Index Mundi United States poverty rate by state 2013 https:/www.indexmundi.com/facts/united-states/quick-facts/virginia/percent-of-people-of-all-ages-in-poverty#map

[14] Richard R Furler J Densley K Equity of access to primary healthcare for vulnerable populations: The IMPACT international survey of innovations Internatl J Equity Hlth 2016 15 1 20 10.1186/s12939-016-0351-7 PMC4828803 27068028

[15] Martsolf G Sloan J Villareal A Mason D Sullivan C Promoting a culture of health through cross-sector collaborations Health Promot Pract 2018 19 5 785 91 10.1177/1524839918772284 29699427

[16] Myers CR Using telehealth to remediate rural mental health and healthcare disparities Issues Ment Health Nurs 2019 40 3 233 9 30508400 10.1080/01612840.2018.1499157

[17] Borges Da Silva R Brault I Pineault R Chouinard MC Prud’homme A D’Amour D Nursing practice in primary care and patients’ experience of care J Primary Care Comm Hlth 2018 9 1 7 10.1177/2150131917747186 PMC5937150 29357748

